# Patchy Dermal Melanocytosis: Differential Diagnosis and Management

**DOI:** 10.1111/jocd.16607

**Published:** 2024-11-01

**Authors:** Jiafang Zhu, Qingqing Cen, Rui Chang, Yue Han, Xiaoxi Lin

**Affiliations:** ^1^ Department of Laser and Aesthetic Medicine, Shanghai Ninth People's Hospital Shanghai Jiao Tong University School of Medicine Shanghai China; ^2^ Department of Dermatology, Shanghai Ninth People's Hospital Shanghai Jiao Tong University School of Medicine Shanghai China; ^3^ Department of Plastic and Reconstructive Surgery, Shanghai Ninth People's Hospital Shanghai Jiao Tong University School of Medicine Shanghai China

**Keywords:** dermal melanocytosis, laser therapy, Mongolian spot, nevus of Ito, patchy melanocytosis, photomechanical effect, photothermal effect

## Abstract

**Background:**

Nevus of Ito and Mongolian spots are distinct clinical presentations of patchy dermal melanocytosis, characterized by similar dermatological manifestations that can pose diagnostic difficulties for clinicians.

**Aim:**

This review aims to consolidate current understanding and research advancements on these conditions to facilitate clinical diagnosis, differential diagnosis, and management.

**Methods:**

A comprehensive search of databases including PubMed and Google Scholar was conducted, along with an analysis of pertinent literature retrieved from reference lists spanning nearly four decades.

**Results:**

Epidemiological, clinical, and pathological profiles exhibit nuanced differences between the two conditions, with unique expressions under electron microscopy and the regression possibility. It is noteworthy that most Mongolian spots naturally fade with advancing age, in contrast to nevus of Ito, which persist and may potentially evolve into malignant lesions. While picosecond laser treatment has shown greater efficacy than nanosecond lasers, the lower‐energy approach holds particular promise in pediatric cases. The therapeutic landscape for patchy dermal melanocytosis is evolving, shifting from selective photothermal action to photomechanical or subcellular photothermal modalities.

**Conclusion:**

This review underscores the importance of meticulous clinical assessment and the potential of innovative therapeutic approaches in managing these conditions.

## 1 Introduction

Mongolian spots, nevus of Ota, and nevus of Ito are all classified under the category of dermal melanocytosis (DM). Clinically, DM presentations can be categorized into two primary series, blue nevus (BN) typically manifests as papular or nodular lesions, whereas the other DMs predominantly present as patchy lesions. The latter category is further classified based on the anatomical location of the lesion: Mongolian spots (MS) in the sacral area or lumbar area, nevus of Ota in the trigeminal nerve area, and nevus of Ito in the acromion‐deltoid area or posterior supraclavicular nerve area [1]. However, Mongolian spots can also occur in the upper limb area, which is known as ectopic (abnormal) Mongolian spots, making it difficult to differentiate it from nevus of Ito. Additionally, the literature documents numerous variants of Mongolian spots and nevus of Ito, as well as case reports with unique clinical features, complicating the diagnosis and differential diagnosis of these two DMs. Large DMs can distress patients psychologically due to cosmetic concerns (Table 1). Laser therapy, which selectively targets dermal melanocytes, has demonstrated efficacy; however, temporary changes in pigmentation, such as hyperpigmentation or hypopigmentation, are frequent adverse effects. Shortening treatment pulse width and reducing energy to minimize adverse reactions continue to guide the direction of laser therapy. This article reviews the differential diagnosis of two DMs with similar presentations: Mongolian spots and nevus of Ito, as well as the treatment advancements for DMs.

**TABLE 1:** Comparison of Mongolian spots and Ito nevus characteristics.

	Epidemiology	Clinical manifestation	Histopathology	Ultrastructure	Regression	Malignant possibility
Mongolian spot	High incidence (90%–100%) M = F	Occur anywhere in the body and are not distributed along the nerves; blue‐green to blue‐gray.	Cell exhibit diverse morphologies and are located in the lower one‐third of the dermis; dermis is unaffected.	The extracellular sheath measures < 1.5 μm and may regress with advancing age.	Will regress in 90% of patients	No reports
Nevus of Ito	Low incidence F > M	Present unilaterally along the posterior supraclavicular nerve; Bilateral distribution occasionally. Yellow‐brown appearance occasionally.	Cell exhibit spindle shape and are located in the upper two‐thirds of dermis; stromal fibrotic reaction may present.	The extracellular sheath measures up to 3 μm thickness and may progress with age	Will not regress and progress occasionally in puberty or pregnancy	Cases were reported

## 2 Methods

This article is conceptualized as a narrative review, aiming to synthesize the existing literature on a range of topics within dermal melanocytosis and its associated conditions. A comprehensive literature search was conducted in PubMed and Google Scholar, scanning the complete databases from their inception up to April 19, 2024. The search terms were crafted to capture a broad spectrum of relevant topics, including “dermal melanocytosis” or “dermal dendritic melanocytic proliferations,” “Mongolian spots” or “congenital dermal melanocytosis,” “nevus of Ito,” “nevus of Ota,” and terms associated with laser treatment for dermal melanocytosis. Additionally, keywords and subject terms were selected to ensure an accurate and thorough exploration of the subject matter. To supplement the database search, a manual perusal of the reference lists of the included articles was also conducted to uncover any additional studies that may have escaped electronic retrieval.

### 2.1 Embryology of Melanocyte

DM occurs when cells differentiate from the neural crest cells and migrate to specific epidermal locations during embryonic development, but become trapped in the dermis, leading to the presence of dendritic melanocytes in the dermis [2]. In humans, melanocyte migration begins at 2.5 weeks of pregnancy and is completed within 8 weeks. The molecular mechanisms of melanocyte migration are not yet fully understood, but it is believed to involve adhesive molecules such as cadherins, integrins, and extracellular matrix elements [3]. Transcription factors such as Sox10, Pax3, FoxD3, and Mitf may be responsible for regulating melanocytes derived from the neural crest, while endothelins‐ligand such as c‐kit (SCF stem cell factor), Wnt proteins, and neuregulin may further regulate melanocyte migration [4]. The number of dermal dendritic cells decreases to some extent after birth and is confined to certain areas of the dermis. They are morphologically different from typical melanocytic nevi (nevocytes) that have completed normal migration and tend to form nests without dendrites [5].

### 2.2 Differentiation Between Mongolian Spot and Nevus of Ito

#### 2.2.1 Epidemiology

Mongolian spots (MS), first proposed by German professor Edwin Baelz in 1885 [5], were considered by many scholars to be inadequate, due to the birthmarks being also reported in non‐Mongolian populations such as Caucasians [6]. However, a group of scholars, led by Zhong, Huang, Nambudiri have proposed renaming the condition as “congenital dermal melanocytosis” to minimize any negative racial connotations [7]. This is due to the higher presence of melanocytes in the skin of Asian and African newborns, making Mongolian spots more likely to occur with an incidence rate as high as 90%–100% [8]. The incidence of Mongolian spots is the same in both males and females. It is worth noting that Nevus of Ito was later reported than Mongolian spots and was first described by Ito in 1954 [9]. This nevus is much rarer than Mongolian spots, with an incidence rate in the Asian population that is < 0.1%. Additionally, it is more prevalent in females than males.

#### 2.2.2 Clinical Manifestations

Mongolian spots, due to their deeper distribution in the dermis, generally appear as blue‐gray or bluish‐black in color. There are several special clinical manifestations of Mongolian spots, including: (1). Superimposed Mongolian spots: A darker Mongolian spot overlies a lighter one; (2). Halo‐like Mongolian spots: When pigmented patches or melanocytic nevi are present in the Mongolian spot area, a white rim lacking epidermal melanocytes locates surrounding each lesion; (3). Speckled Mongolian spots: It appears as groups of dotted pigmentation [10, 11, 12, 13]. The overall color of Mongolian spots is relatively uniform, with blue‐green or blue‐gray appearance, and is determined by the Tyndall Effect. Nevus of Ito generally appears more heterogeneous in color [14] and may be related to the presence of unpigmented melanocyte precursors. However, because melanocytes distribute in the superficial dermis, they may appear somewhat yellow‐brown in color. The size of Mongolian spots can vary greatly, ranging from a few centimeters to over 20 cm. In contrast, nevus of Ito generally has a relatively fixed size range.

One of the major differences between Mongolian spots and nevus of Ito is that Mongolian spots do not follow the nerve distribution. They are predominantly found on the lumbosacral region, accounting for approximately 80.63% [8]. Other affected sites include the lower limbs, upper back, upper limbs, perineum, shoulders, and chest [15]. Mongolian spots that do not occur in the lumbosacral region are also named aberrant MS. Mongolian spots that are distributed on the upper limbs may be confused with nevus of Ito. However, nevus of Ito is fixedly distributed in the region at the posterior supraclavicular nerve and the lateral cutaneous nerve of the upper arm, with no report of involvement of other nerves, such as the lateral antebrachial cutaneous nerve to date. Occasionally, it may involve one side of the neck, the scapula area, and the shoulder joint region [16]. Nevus of Ito, like nevus of Ota, also has reports of bilateral symmetrical distribution [14].

#### 2.2.3 Histopathology

Histopathology is the gold standard for differential diagnosis, and there are subtle differences between Mongolian spots and nevus of Ito in histopathology. Mongolian spots have melanocytes distributed in the middle to lower layers of the dermis, with various cell shapes ranging from bipolar, pear‐shaped, elliptical, or irregular. No accompanying stromal fibrotic reaction is present [17]. Nevus of Ito, on the other hand, are located in the upper to middle layers of the dermis [18], and the cells can take on a spindle‐shaped appearance, or bipolar dendritic melanocytes with their axis aligned parallel to the skin surface and possibly accompanied by a stromal fibrotic reaction [19].

#### 2.2.4 Ultrastructure

Under electron microscopy, dermal melanocytes are observed to contain abundant melanosomes, which are enveloped by extracellular sheaths. These sheaths are well developed in nevus of Ito and are composed primarily of fine granules and filaments, with a granular component dominance. The width of the extracellular sheaths can reach up to 3 μm, approximately double the width of the Mongolian spots cell sheath. It is estimated that the cell sheath occupies up to 97% of the melanocyte area. No significant ultrastructural changes were observed in the melanocyte sheaths of nevus of Ito, irrespective of the patients' age differences (39 and 64 years) [20].

In Mongolian spots, the melanocyte sheath is notably less developed, with maximum 1.5 μm characterized by a predominantly filamentous texture. As the patient ages (from 2 months to 6 years), there is a significant regression in the development of the cell sheath. A common observation in Mongolian spots is a partial disruption of the cytolemma surrounding the dermal melocytes, which can lead to the release of melanosomes into the connective tissue matrix, potentially affecting the pigmentation degree within the skin [20].

#### 2.2.5 Regression

A salient differentiating characteristic between Mongolian spots and nevus of Ito is the fade pattern observed over time. Mongolian spots typically exhibit a progressive fading process, with most beginning to diminish around the first year of life and vanishing entirely by approximately the sixth year. Those that persist into adulthood, showing no signs of fading, are categorized as persistent Mongolian spots. In stark contrast, nevus of Ito do not fade spontaneously and may even deepen in coloration following the onset of puberty.

The prevalence of persistent Mongolian spots is relatively rare, with an estimated probability of 2.8%–4.1% [13]. Epidemiological studies suggest that Mongolian spots with darker pigmentation, a higher number, located beyond the sacral region, and larger than 10 cm in diameter have a higher likelihood of persisting. Newborns with significant, non‐fading Mongolian spots may be associated with metabolic disorders, including mucopolysaccharidosis and gangliosidosis GM1 [21, 22, 23, 24]. In cases where metabolic abnormalities are present, the Mongolian spots may not only be generalized around the sacrum but can also extend abnormally to the back, legs, abdomen, and include abnormal hair growth [21, 22]. The accumulated metabolic substances in these conditions bind to the Trk protein, resulting in an abnormal increase in the activity of nerve growth factor (NGF), which binds to the tyrosine kinase receptor and promotes the abnormal migration of melanocytes [21].

Vascular malformations are sometimes associated with persistent, non‐fading Mongolian spots. The most prevalent of these is Phacomatosis Pigmentovascularis (PPV). PPV encompasses five distinct clinical subtypes: Type I, characterized by capillary malformations with pigmented linear epidermal nevus; Type II, featuring capillary malformations with dermal melanocytosis, with or without anemic nevus; Type III, presenting with capillary malformations and nevi spilus, with or without anemic nevus; Type IV, which includes capillary malformations and dermal melanocytosis, with or without anemic nevus; and Type V, defined by cutis marmorata telangiectatica congenita with dermal melanocytosis [25]. To date, patients with Types II, IV, and V have been documented with Mongolian spots and nevus of Ota, but there have been no reported cases of nevus of Ito. The development of PPV is thought to be linked to genetic mutations in the GNA11 or GNAQ genes [26]. These mutations can compromise the functional activity of the GTPase, leading to the persistent activation of the downstream signaling pathway and subsequent abnormal pigment expression [26, 27].

Contrary to Mongolian spots and other vascular malformations, there is no known association between nevus of Ito and metabolic abnormalities or vascular malformations. Similar to nevus of Ota, nevus of Ito has the potential to progress with advancing age. When nevus of Ito gradually presents with deep irregular masses, in addition to routine diagnosis and differential diagnosis with common conditions such as sebaceous cysts, one should also be alert with the possibility of malignant melanoma [28]. The differences between Mongolian spots and nevus of Ito are summarized in Table 1.

### 2.3 Progress in Laser Therapy for DM

#### 2.3.1 Treatment Age

The timing of treatment for DM is crucial, with a strong recommendation to initiate laser therapy before the age of 5. Early laser intervention at this age range can facilitate more rapid achievement of therapeutic goals [29]. Moreover, given that DM can be present on body‐exposed areas such as the face, arms, or legs, completion of treatment before the age of 5 can significantly benefit patients by mitigating future social stress associated with the condition.

#### 2.3.2 Treatment Interval

It is advisable to maintain a treatment interval of > 6 months for DM treatment. Prolonging the interval between treatments can help mitigate the risk of post‐inflammatory hyperpigmentation. Additionally, this approach allows for more effective phagocytosis and metabolism of the destroyed melanocytes by macrophages, potentially reducing the overall number of treatment sessions and lessening the economic and psychological burden on patients [30].

### 2.4 Treatment Methods

#### 2.4.1 Q‐Switched 755 nm Laser

The 755 nm wavelength of the Q‐switched laser aligns with the medium absorption peak of melanin, enabling deeper penetration of more than 0.5 mm into the dermis for enhanced treatment of hyperpigmentation. The 70 ns pulse width of the Q‐switched 755 nm laser, compared with the thermal relaxation time of melanosomes being between 15 and 150 ns [31], theoretically allows for the effective targeting of melanosomes without significantly impacting the surrounding tissue. In the treatment of Mongolian spots with the Q‐switched alexandrite laser (ALEXLAZR; Candela, Wayland, MA), using a 4 mm spot size, a 755 nm wavelength, and a pulse duration of 4–5 J/cm^2^, the overall effective rate was 75%, with a hyperpigmentation incidence of 31.25% after 3–6 sessions [32].

Considering the higher occurrence of post‐inflammatory hyperpigmentation in East Asian patients, reducing treatment energy and increasing spot size can enhance laser penetration and mitigate post‐operative hyperpigmentation and other adverse effects, making this approach more suitable for pediatric patients under the age of 5. Wu et al. compared the efficacy of the Q‐switched 755 nm alexandrite laser (Accolade; Cynosure, Westford, MA) with a 5 mm spot size, 2.2–2.8 J/cm^2^ energy, and 10 Hz speed to the traditional 3 mm spot with 5.0–8.0 J/cm^2^ energy and 2.5 Hz speed. They confirmed that the larger spot size and lower energy setting can more effectively reduce hyperpigmentation, with superior outcomes observed after 3 treatments. These findings imply that the low energy mode of treatment may have broader applicability in the management of DM [33].

#### 2.4.2 Picosecond 755 nm Laser

The picosecond 755 nm laser operates with a pulse width that is abbreviated to between 550 and 750 ps. Given that this pulse duration is substantially shorter than the thermal relaxation time of melanosomes, it is theorized that the incidence of side effects during treatment is correspondingly decreased. The photomechanical effect generated by picosecond lasers further fragments melanosomes, thereby augmenting the treatment's effectiveness. Ge et al. retrospectively evaluated the treatment outcomes of 56 patients with DM who were treated with the picosecond 755 nm laser, utilizing parameters of a 2.0–4.0 mm spot size, 1.59–6.37 J/cm^2^, and a 5 Hz frequency. In comparison to the Q‐switched 755 nm laser with a 3.0 mm spot size, 5.0–7.0 J/cm^2^, and a 5 Hz frequency, the picosecond laser demonstrated enhanced efficacy and significantly reduced rates of pain, post‐inflammatory hyperpigmentation, and hypopigmentation [34]. Concurring findings were reported by Imagawa et al. and Ohshiro et al., underscoring the safety and efficacy of the picosecond 755 nm laser for DM treatment [35, 36].

#### 2.4.3 P730 nm and P785 nm Lasers

Both the P730 nm and P785 nm lasers are classified as picosecond devices, sharing absorption peaks akin to that of the P755 nm laser. These lasers boast even shorter pulse widths and have demonstrated promising application potential based on recent case studies. Tiffany et al. successfully treated a DM lesion using the P730 nm laser, with a pulse width of 250 ps, a 4.0 mm spot size, 1 J/cm^2^, and four passes, repeated once a month for four treatments. Another case involving the P785 nm laser, with a 300 ps pulse width, 4.0 mm spot size, 1 J/cm^2^, and four passes, repeated once a month for three treatments, improved a nevus of Ota that had previously been unresponsive to P755 nm and P1064nm laser treatments [37]. Notably, both treatments adopted a low‐energy, high‐frequency strategy, differing from the conventional high‐energy, low‐frequency approach. This methodological shift effectively truncates the overall treatment duration and significantly mitigates the side effects associated with individual treatment sessions.

#### 2.4.4 Q1064nm Laser

The treatment window for Q‐switched 755 nm laser therapy is broader than that for Q‐switched 1064 nm laser treatment, although the Q‐switched 1064 nm laser offers deeper penetration into the skin. This deeper penetration theoretically makes it safer for patients with darker skin tones by minimizing epidermal damage [38, 39]. However, it is considered that when the Q1064 nm laser is employed to treat DM in light‐skinned patients, its efficacy may be inferior to that of the Q755 nm laser due to the reduced melanin absorption at the 1064 nm wavelength. Choi et al. conducted a comparative study using the Q755 nm laser (Candela Corporation, Wayland, MA) with energy densities ranging from 5.5 to 8.0 J/cm^2^ and the Q1064 nm laser (Candela Corporation, Wayland, MA) with energy densities of 6 J/cm^2^–12 J/cm^2^ for treating nevus of Ota. The results indicated that the Q755 nm laser outperformed the Q1064 nm laser in terms of efficacy [40].

For young patients with DM, the efficacy of treatment can be significantly decreased when intravenous anesthesia is not administered. The discomfort and fear associated with the procedure can result in incomplete treatment of lesions, which in turn leading to a mottled skin appearance. Furthermore, the need for high‐energy lasers necessitates treatment intervals of at least 6 months, which can extend the overall treatment duration to approximately 2 years, thereby increasing the psychological and emotional stress on patients and their families [41]. Recently, researchers have advocated for the use of 1064 nm laser therapy with relatively low energy and a larger spot size for young children under the age of 5. This approach inflicts a sublethal injury on melanocytes instead of destruction, thereby minimizing epidermal and subcutaneous tissue damage in the child [42]. Moreover, the treatment interval is reduced to 2 weeks, offering patients and their families a more expedient resolution of the pigmented appearance. Shin et al. utilized low‐energy Q1064 nm laser treatment with a 7 mm spot size, 2.0–2.4 J/cm^2^ energy, and a 10 Hz speed. The treatment endpoint was characterized by slight skin redness and petechiae. Sessions were scheduled once a month, with an average patient age of 16.2 months (range: 2–84 months). The average number of laser treatments was 10.3 ± 4.9, and the median treatment duration was 14.1 months. The incidence of adverse reactions was 6.1%, and the level of pain experienced was minimal. These findings suggest that repetitive low‐energy Q1064 nm laser therapy may hold promise for the treatment of extensive dermal melanocytosis in young children [43].

#### 2.4.5 P1064 nm Laser

The P1064 nm laser, characterized by a shorter pulse width than its Q‐switched counterpart, has the potential to mitigate treatment‐related complications. Furthermore, the P1064 nm laser's ability to penetrate deeper into the skin exceeds that of the P755 nm laser, theoretically enhancing the safety profile when treating DM in patients with darker skin tones. Yang et al. utilized a 3–4 mm spot size, 1.8–4.3 J/cm^2^ energy, and a 5 Hz frequency, with the endpoint response indicating pinpoint bleeding. Treatment sessions were scheduled from 1 to 5 times. Following a single treatment, patients achieved an efficacy score of 2.56, which was higher than that reported for the Q1064 nm laser but lower than the efficacy observed with the P755 nm laser. Nonetheless, the P1064 nm laser exhibited a lower incidence of adverse effects at just 6.25%, which is less than the complication rate observed with the P755 nm laser. These findings suggest that the P1064 nm laser may offer a safety advantage in the treatment of dermal melanocytosis [44].

## 3 Conclusions

The discrimination between Mongolian spots and nevus of Ito may be achieved through a variety of approaches, which include but are not limited to epidemiological analysis, clinical manifestations, histopathological examination, and electron microscopic evaluation. Despite these distinctions, the precise reasons why Mongolian spots may fade whereas nevus of Ito may progress are not fully understood. Additional research, employing histopathological and genetic testing, is required to elucidate the underlying mechanisms. In the realm of treatment for dermal melanocytosis, targeted gene therapy may emerge as a promising alternative to laser therapy in the future. This novel approach would aim to reduce the number of dermal melanocytes and diminish the treatment‐related downtime experienced by patients. Currently, the treatment landscape for congenital dermal melanocytosis is evolving, shifting from selective photothermolysis to photomechanical effects or subcellular photothermolysis. However, there is a paucity of large‐scale, self‐controlled studies that comparatively assess the efficacy and safety of various picosecond lasers versus low‐energy, high‐frequency laser treatments. Future controlled studies that encompass diverse age groups, skin colors, and stratified analyses are essential. Such research will enhance our comprehension of the benefits and limitations of different therapeutic modalities, thereby enabling us to more adeptly determine the indications for each treatment option.

## Conflicts of Interest

The authors declare no conflicts of interest.

## Data Availability

Data sharing not applicable to this article as no datasets were generated or analysed during the current study.
